# Activity-dependent expression of neuronal PAS domain-containing protein 4 (npas4a) in the developing zebrafish brain

**DOI:** 10.3389/fnana.2014.00148

**Published:** 2014-12-04

**Authors:** Thomas Klarić, Michael Lardelli, Brian Key, Simon Koblar, Martin Lewis

**Affiliations:** ^1^School of Molecular and Biomedical Sciences, The University of AdelaideAdelaide, SA, Australia; ^2^School of Biomedical Sciences, The University of QueenslandBrisbane, QLD, Australia; ^3^School of Medicine, The University of AdelaideAdelaide, SA, Australia

**Keywords:** npas4a, zebrafish, neurodevelopment, dlx1, shh, PTZ

## Abstract

In rodents, the Npas4 gene has recently been identified as being an important regulator of synaptic plasticity and memory. Homologs of Npas4 have been found in invertebrate species though their functions appear to be too divergent for them to be studied as a proxy for the mammalian proteins. The aim of this study, therefore, was to ascertain the suitability of the zebrafish as a model organism for investigating the function of Npas4 genes. We show here that the expression and regulation of the zebrafish Npas4 homolog, npas4a, is remarkably similar to that of the rodent Npas4 genes. As in mammals, expression of the zebrafish npas4a gene is restricted to the brain where it is up-regulated in response to neuronal activity. Furthermore, we also show that knockdown of npas4a during embryonic development results in a number of forebrain-specific defects including increased apoptosis and misexpression of the forebrain marker genes dlx1a and shha. Our work demonstrates that the zebrafish is a suitable model organism for investigating the role of the npas4a gene and one that is likely to provide valuable insights into the function of the mammalian homologs. Furthermore, our findings highlight a potential role for npas4a in forebrain development.

## Introduction

Genes with important functions, such as those that are crucial for an organism’s survival or those that contribute to its overall fitness, tend to be highly conserved across vast evolutionary distances due to the strong selective pressure to retain that particular function. Thus, it is often possible to gain insights into the function of a gene by studying its homolog in a closely related, but more tractable, species. It is this principle that has allowed humble organisms like the worm and the fruit fly to become the workhorses of modern molecular genetics. Even in seemingly complex biological processes, such as embryo segmentation, much of the initial progress into understanding the function of genes involved in these processes occurred in these relatively simple organisms (Heffer and Pick, 2013).

Recently, the mammalian Npas4 genes have been identified as being important regulators of synaptic plasticity (Lin et al., 2008; Bloodgood et al., 2013) and memory (Ploski et al., 2011; Ramamoorthi et al., 2011) as a result of some ground-breaking studies performed in rodents. These genes encode activity-dependent (Xiang et al., 2007; Lin et al., 2008; Bepari et al., 2012a,b) basic helix-loop-helix (bHLH) Per-Arnt-Sim (PAS) transcription factors (Moser et al., 2004; Ooe et al., 2004; Hao et al., 2011; Pruunsild et al., 2011) that are expressed predominantly in the brain (Moser et al., 2004; Ooe et al., 2004; Shamloo et al., 2006; Hester et al., 2007). Though deletion of Npas4 is not lethal, mice lacking the Npas4 gene display numerous deleterious phenotypes that negatively impact upon the animal’s fitness including a shortened lifespan (Ooe et al., 2009), vulnerability to seizure (Lin et al., 2008), memory impairments (Ramamoorthi et al., 2011; Coutellier et al., 2012) and sensorimotor deficits (Coutellier et al., 2012). Thus, given the obvious importance of these genes in neuronal function, one might expect that their function would be highly conserved throughout evolution.

Indeed, putative homologs of the Npas4 genes have been identified in both the fruit fly *Drosophila melanogaster* (Jiang and Crews, 2003) and the nematode *Caenorhabditis elegans* (Ooe et al., 2004) where they are known as dysfusion (dys) and C15C8.2 respectively, though it appears that there has been considerable divergence in the function of the Npas4-related genes since the branching of vertebrates and invertebrates. Based on amino acid homology within the conserved amino-terminal bHLH domain, the human NPAS4 protein and the invertebrate Npas4-like factors are more closely to each other than to other bHLH PAS factors within the same species (Ooe et al., 2007) and hence these proteins cluster together to form a distinct subgroup within the bHLH PAS family (Jiang and Crews, 2003). This suggests that all of the Npas4-related genes are derived from a common ancestral gene which existed before the divergence of nematodes, insects and vertebrates. Nevertheless, in the carboxy-terminal portion of the proteins, where the transactivation domain is located, the amino acid sequences are poorly conserved (Ooe et al., 2004, 2007). Therefore, rather than being true orthologs, it is possible that the Npas4-like factors have acquired non-conserved functions throughout the course of evolution and the current evidence suggests that this is the case.

While there are many parallels between the Npas4-related genes, there are also crucial differences. All of the Npas4-related genes studied thus far encode transcription factors that act as transcriptional activators (Ooe et al., 2007). Indeed, reporter gene assays have shown that there has been a substantial amount of conservation in the basic biochemical properties of the Npas4 homologs throughout evolution. Like their mammalian counterparts, the invertebrate homologs do not homodimerize, but are capable of binding their respective aryl hydrocarbon receptor nuclear translocator (Arnt) orthologs to form a transcriptionally-active complex (Jiang and Crews, 2007; Ooe et al., 2007). Furthermore, these heterodimers are able to bind the NCGTG DNA motif that is recognized by the vertebrate Npas4 homologs (Jiang and Crews, 2007; Ooe et al., 2007). Nevertheless, despite these fundamental mechanistic similarities, the expression and functional data that are available indicate that the vertebrate and invertebrate homologs are very different from each other on a functional level.

Both of the invertebrate Npas4 homologs that have been studied are expressed in tubular structures of the feeding and respiratory systems. In the fruit fly, the dys gene is expressed in tracheal fusion cells (specialized cells located at the tips of tracheal tubules) where it controls the process of tracheal branch fusion (Jiang and Crews, 2003, 2006). In the nematode, the C15C8.2 protein, also referred to as cky-1 or *C. elegans* NXF-like-factor (cNXFL), is expressed in the pharynx (Crews, 2003) which raises the possibility that it may have a similar role to Dys in regulating the development of hollow tube-like structures though, as yet, there have been no studies conducted to determine the function of this protein. Based on this evidence, it seems that the vertebrate and invertebrate Npas4 proteins have diverged significantly enough in their expression patterns and functions that it would be futile to use one as a model for the other if the goal is to understand their biological role.

For this reason, a model organism that is evolutionarily closer to mammals may be a more suitable vehicle for probing the function of the Npas4 genes. Curiously, despite the many advantages offered by lower vertebrates such as birds, frogs and fish, there has been minimal research into the nature of the Npas4 homologs beyond rodents and humans. While great gains have been made in understanding the function of Npas4 in rodents, some areas of research remain poorly characterized. For instance, little is known about the expression of the Npas4 genes during embryogenesis and whether or not they play a role in embryonic development. One study has demonstrated that in the mouse embryo Npas4 is expressed in the developing forebrain (Klaric et al., 2014), though the functional significance of this expression has not been investigated. The limited amount of data on this subject may be a result of the inherent difficulties of investigating embryonic development in mammals and for this reason certain species may be better suited to addressing these types of questions. One such species is the zebrafish *Danio rerio*, a widely used vertebrate model organism with great utility in defining neuro-developmental processes due to the transparency of the embryos coupled with their external development.

It has been shown that many important genes have been found to have substantially similar functions in fish and mammals (Scholpp and Lumsden, 2010; Hagemann and Scholpp, 2012; Andoniadou and Martinez-Barbera, 2013). We reasoned, therefore, that the zebrafish might be a more appropriate model organism in which to study the function of the mammalian Npas4 genes than the invertebrate homologs due to the higher degree of conservation that is found among vertebrate homologs. In particular, we reasoned that the zebrafish would provide an excellent platform for probing the possible role of Npas4 genes in embryonic development, an area of research that has been largely overlooked in mammals thus far. In light of this, the aim of this study was to perform an initial characterization of the *D. rerio* Npas4 homolog, npas4a, to determine its suitability as a proxy for the mammalian genes. We first examined the evolutionary relatedness of the zebrafish npas4a protein with respect to other Npas4 homologs. Next, we investigated the regulation and expression of the npas4a gene at the transcript level at various time-points during development. Finally, we exploited the inherent advantages of the zebrafish model to explore the function of the npas4a gene in central nervous system (CNS) development by using loss-of-function experiments in the developing embryo.

## Materials and methods

### Animals

Experiments were performed with the approval of the University of Adelaide Animal Ethics Committee and in accordance with Research Ethics and Compliance Unit guidelines (Permit numbers S03302006 and S2009070). The Tübingen strain of zebrafish was used for all experiments. Embryos were collected from our breeding colony and allowed to develop at standard temperature (28.5°C) (Kimmel et al., 1995). Collected embryos were maintained in fresh Embryo Medium (13.72 mM NaCl, 0.54 mM KCl, 0.25 mM Na_2_HPO_4_, 0.44 mM K_2_HPO_4_, 1.0 mM CaCl_2_, 1.0 mM MgSO_4_, 4.2 mM NaHCO_3_). Embryos collected prior to hatching were dechorionated before use. Those collected from 48 h post-fertilization (hpf) onwards were treated with 40 μM 1-phenyl-2-thiourea to prevent pigmentation.

### Phylogenetic analysis

Pairwise global alignments were performed using the National Centre for Biotechnology Information (NCBI) Protein Blast tool. A phylogenetic tree was created using PhyML as previously described (Dereeper et al., 2008).

### Pentylenetetrazol (PTZ) treatment

Embryos: Twenty-four or 72 hpf embryos were placed in embryo medium containing 3.3 mM PTZ for 20 min and were then allowed to recover in fresh embryo medium for 30 min at which time they were fixed for gene expression analysis. Control fish were treated as above but were placed in embryo medium that did not contain PTZ.

Adult fish: One year old zebrafish were placed in tank water containing 3.3 mM PTZ for 20 min and were then allowed to recover in fresh tank water for 60 min before their brains were removed for RNA extraction. Control fish were treated as above but were placed in tank water that did not contain PTZ. Seizures were identified when zebrafish displayed uncontrolled movements and rapid swimming. Loss of conscious was noted when, following seizure, the zebrafish rolled over with ventral side upwards and were immobile for a period of approximately 10 s before righting themselves and resuming swimming.

### Quantitative reverse-transcription polymerase chain reaction (qRT-PCR)

RNA was extracted from the brains of PTZ-treated adult zebrafish using TRIzol (Life Technologies) and then further purified using an RNeasy (QIAGEN) silica-based spin column. Complementary DNA was synthesized from the RNA using Superscript III (Life Technologies). Amplification by qRT-PCR was performed using TaqMan master mix and an ABI PRISM® 7000 Sequence Detection System driven by ABI PRISM SDS v1.1 software (Applied Biosystems®) was used for thermal cycling. The zebrafish β-actin ortholog, actb2, was used as an internal reference to facilitate relative quantification using the comparative Ct method (Schmittgen and Livak, 2008). Primers directed to the npas4a transcript were similar in reaction efficiency to the actb2 internal reference primers. Oligonucleotides were ordered from GeneWorks, Australia. Primer sequences: npas4a 5′-AGCCAAGTCTGCCCTTCTTCT-3′ and 5′-TGCTGTGCTAAAAGCGAGATCT-3′ (76 bp product), actb2 5′-GGTATGTGCAAAGCCGGATT-3′ and 5′-ACCAACCATGACACCCTGATG-3′ (96 bp product). Samples were loaded in triplicate.

### Morpholino oligonucleotide (MO) injections

An oligonucleotide targeting the 5′ untranslated region (5′ UTR) of the npas4a transcript was constructed with the sequence: 5′-GCAACGATGAAAACTGTTTCTGAGC-3′. The sequence of the mismatch control morpholino oligonucleotide (MO; with substitutions underlined) is as follows: 5′-GCAAGGATCAAAAGTGTTTGTCAGC-3′. A BLAST search of the zebrafish genome database (Zv9) verified that the target site of the MO was specific to the npas4a transcript. As far as possible, MO experiments were performed to the standards and conventions recommended by our peers (Corey and Abrams, 2001; Eisen and Smith, 2008). All MOs were purchased from Gene Tools, USA. Morpholino oligonucleotides were solubilized in sterile water and diluted to 500 nM. Injection volumes were calibrated and 1.75 pmol delivered into single cell embryos.

### Construction of 5′ UTR MO enhanced green fluorescent protein (eGFP) reporter

The following primers were used to clone the npas4a 5′ UTR upstream of the eGFP coding sequence (5′ UTR MO target sequence underlined): 5′-GGAATTCCTGTTTTCAGTCAATCACCTACAGGAGAGAGAGACCGCTCAGAAACAGTTTTCATCGTTGCCCCTCATGGTGAGCAAGGGCGAGGAG-3′ and 5′-GGCATGGACGAGCTGTACAAGTAATTAATTAATCTAGACC-3′. The PCR product was propagated in the pGEM®-T Easy (Promega) vector, excised with SacII and XbaI and inserted after the CMV promoter in the pEGFP-N1 vector (accession U55762) which was also cut with SacII and XbaI.

### *In situ* hybridization (ISH)

To construct the npas4a ISH probes, a 587 bp region of DNA from exon 7 of the npas4a transcript was amplified using the following primers: 5′-CCTCTTCCACCTGTGCCTAC-3′ and 5′-CCCTCTGCTAGTGTACTGATG-3′. This fragment was cloned into the pGEM®-T plasmid (Promega) and riboprobes (sense: SP6, anti-sense: T7) were labeled with digoxigenin-11-dUTP (Roche). Free label was removed by diluting to 0.5 mL and passing through a Nanosep® 30 K column at 5.0 g for 12 min and resolubilizing in 40 μL diethylpyrocarbonate (DEPC) treated water. Riboprobes for pax6a (sense: T3, anti-sense: T7) were produced by in a pBluescript SK plasmid as described by Nornes et al. (1998). Riboprobes for fgf8a (sense: T3, anti-sense: T7) were made from 2.5 kb inserts cloned into the pBluescript SK vector and then linearized with XbaI. Similarly, riboprobes for dlx1a (sense: T7, anti-sense: T3) and shha (sense: T3, anti-sense: T7) were linearized with HindIII. Hybridizations were performed at the nominated time points as described by Jowett (1997).

Dechorionated embyros were fixed in 4% w/v paraformaldehyde (PFA) and stored at 4°C until hybridization. Whilst rocking embryos were rinsed with then placed in 100% fresh methanol for 2 h at −20°C, then 5 min each in 75%, 50% and 25% methanol/PBS followed by four 5 min washes in 0.1% Tween20 in PBS (PBST). This was followed by Proteinase K treatment (20 mg/mL in PBST at 37°C) for either 5 min (48 hpf embryos) or 10 min (72 hpf embryos). Twenty-four hpf embryos were not treated with Proteinase K. Embryos were then fixed for 20 min in 4% PFA and washed four times for 5 min in PBST. Prehybridization was performed at 70°C for 2 h with gentle rocking in lockable tubes. The prehybridization solution (1 mL per tube) was prepared in DEPC treated water and contained 0.5 mg/mL heparin (Sigma H3393), 0.5 mg/mL yeast RNA (Roche 109223), 0.5% CHAPS (Sigma C3023), 2% blocking reagent (Roche 1096176), 50% deionized formamide (Chemicon 54117), 5 mM EDTA pH 8 and 5× saline sodium citrate (SSC).

Following this, embryos were incubated with fresh prehybridization solution containing riboprobes (300 ng/μL per tube) overnight at 70°C. Embryos were then rinsed three times in 1 mL prehybridization solution, washed twice more in prehybridization solution at 70°C for 15 min and then washed for 30 min at 70°C in a 1:1 mixture of prehybridization solution and 2× SSC. Embryos were then rinsed in 0.2× SSC with 0.1% CHAPS and then washed in the same solution twice for 30 min at 70°C. Next, embryos were rinsed once and washed twice for 10 min in PBST at 25°C.

After this, embryos were rinsed then washed for 2–3 h in 1% bovine serum album (BSA) in PBST at 4°C. Next, embryos were incubated in anti-DIG Fab fragment (Roche; 1:4000 dilution in 1% BSA in PBST) for 1 h at 4°C. Embryos were rinsed three times, then washed five times for 1 h in 0.1% BSA in PBST at 25°C. Embryos were then rinsed once and washed three times for 5 min in NTMT solution (100 mM NaCl, 100 mM Tris pH 9.5, 50 mM MgCl_2_, 0.1% Tween20). Finally, embryos were incubated for 30 min at 25°C in staining solution (3 mL NTMT, 10.2 μL NBT, 10.2 μL BCIP, Roche) or at 4°C until the color reaction was complete.

### Apoptosis and mitotic assays

The protocol for the *in situ* terminal deoxynucleotidyl transferase-mediated dUTP nick-end labeling (TUNEL) assay that was used to measure cell death has been described previously (Cole and Ross, 2001). Briefly embryos were fixed in 4% PFA and the *In Situ* Cell Death Detection Kit (Roche) was to detect apoptotic cells detected by visualization with metal-enhanced 3,3′-diaminobenzidine (DAB; Pierce). The mitosis assay using an anti-phospho-histone H3 antibody (rabbit polyclonal IgG; Upstate Biotechnology; 1:300 dilution) was visualized with DAB peroxidase substrate (Sigma-Aldrich D-4293) as described by Léger and Brand (2002). To aid in visualization, the skin, eyes and yolk sac were removed from the embryos prior to imaging.

### Immunohistochemistry

Immunohistochemistry was performed on whole mount 24 hpf zebrafish embryos as previously described (Hjorth and Key, 2001). Briefly, fixed embryos were washed four times for 5 min in PBS containing 1% BSA and 1% dimethylsulfoxide (PBD), permeabilized in PBST for 16 h at 4°C, washed four times 5 min in PBD at 25°C and then blocked in blocking solution (5% horse serum in PBD) for 3 h at 25°C. Embryos were then incubated in fresh blocking solution containing primary antibody overnight at 4°C with gentle shaking. The rabbit anti-epha4a primary antibody, a gift from David Wilkinson (Mill Hill, UK), was diluted 1:3000 while the mouse anti-elavl3 antibody, a gift from Kirk Jensen (University of Adelaide, Australia), was diluted 1:500. Embryos were then washed in 1 mL PBD five times for 1 h before being incubated with the appropriate alkaline phosphatase conjugated secondary antibody (Jackson; diluted 1:1000 in blocking solution) for 16 h at 4°C with gentle rocking. Following this, embryos were washed five times 1 h with 0.1% Tween20 in PBD. Next, embryos were washed three times for 5 min in NTMT and incubated in staining solution (3.5 μL BCIP and 3.4 μL NBT per 1 mL NTMT with 1 mM levamisole) until the color reaction was complete. After color development, embryos were rinsed then washed for 10 min in NTMT, rinsed then washed in Tris-buffered saline containing 0.1% Tween20 for 10 min, fixed for 16 h at 4°C with 4% PFA and cleared for imaging in 80% glycerol overnight.

## Results

### Evolutionary comparison of Npas4 homologs across species

**Figure 1:**
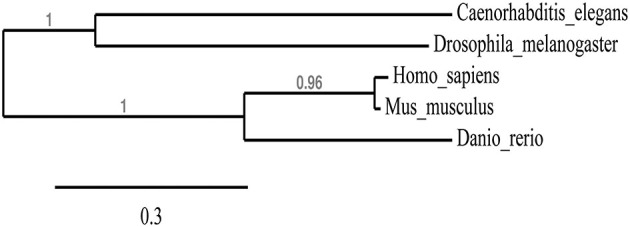
**Phylogenetic tree showing the relationship between Npas4 homologs**. Alignments from Table 1 were used to create a phylogenetic tree of Npas4 proteins using PhyML. Sequences used were as in Table 1.

We first wanted to determine the extent to which the zebrafish npas4a protein is conserved relative to Npas4 homologs in other species. Table 1 shows an alignment of the amino acid sequences of the Npas4 homologs from *Homo sapiens*, *Mus musculus*, *Danio rerio*, *Drosophila melanogaster* and *Caenorhabditis elegans*. Interestingly, the zebrafish protein has a unique 100-residue insert in the transactivation domain 60 amino acids upstream from the carboxy-terminus that is not present in other species. When aligned, the zebrafish npas4a protein shows only a moderate degree of overall conservation with the mammalian Npas4 proteins (<60% identity) and still less with the invertebrate Npas4 proteins (<35% identity) which suggests that the zebrafish homolog has diverged significantly throughout the course of evolution (Table 1). This was evident when a phylogenetic tree was drawn to display the relationship between the different Npas4 homologs as it showed that although the zebrafish npas4a protein was more closely related to the mammalian proteins than the invertebrate proteins, it did not cluster within either of these clades and instead formed its own individual branch (Figure 1). This divergence raised the possibility that the zebrafish npas4a gene may have acquired novel functions that are distinct to those of the mammalian and invertebrate homologs. We therefore decided to investigate the expression, regulation and function of the zebrafish npas4a gene to determine whether it shared any characteristics with the homologous genes from other species or whether it had diverged to such an extent that it has taken on a completely novel role in fish.

**Table 1:** **Npas4 protein sequence homologies between species**.

	*Homo sapiens*	*Mus musculus*	*Danio rerio*	*Drosophila melanogaster*	*Caenorhabditis elegans*
*Homo sapiens*	-	**93**	**58**	**32**	**35**
*Mus musculus*	95	-	**58**	**33**	**34**
*Danio rerio*	71	70	-	**31**	**33**
*Drosophila melanogaster*	48	50	49	-	**39**
*Caenorhabditis elegans*	53	54	53	57	-

*Pairwise global alignments were performed using the NCBI Protein Blast tool to determine the percentage of **global identity** (top right) and similarity (bottom left) between species. Sequences aligned were: H. Sapiens Q7IUM7.1 (802 aa), M. musculus Q7BGD7.1 (802 aa), D. rerio Q1ECW2.1 (933 aa), D. melanogaster Q4V724 (885 aa), C. elegans G5EFL9 (676 aa)*.

### Expression of the zebrafish npas4a gene is restricted to the forebrain during embryonic development

The mammalian Npas4 genes are expressed predominantly in the brain (Moser et al., 2004; Ooe et al., 2004; Shamloo et al., 2006; Hester et al., 2007), while the invertebrate homologs can be found in tubular structures of the feeding and respiratory systems with the *D. melanogaster* homolog dys being expressed in the trachea (Jiang and Crews, 2003, 2006) and the *C. elegans* homolog C15C8.2 being expressed in the pharynx (Crews, 2003). However, in contrast to the wealth of information concerning the expression of these Npas4 genes, little is known about the expression pattern of homologous Npas4 genes in lower vertebrates, such as fish.

**Figure 2:**
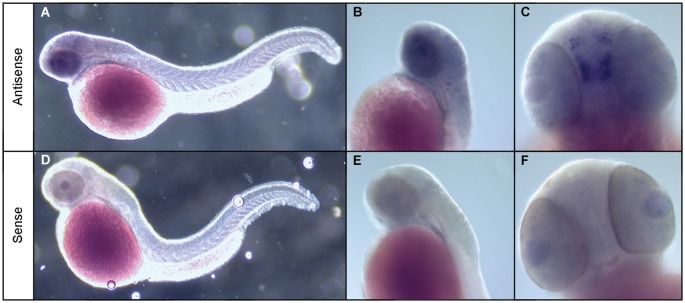
**Expression of npas4a mRNA in the developing zebrafish brain**. Whole-mount ISH of 48 hpf zebrafish embryos with npas4a antisense **(A–C)** and sense **(D–F)** digoxigenin-11-dUTP labeled probes. Representative lateral **(A,B,D,E)** and ventral **(C,F)** views are shown.

To address this, we used whole mount ISH to investigate the expression of the npas4a gene in zebrafish embryos. While we were unable to detect npas4a expression at 24 hpf (data not shown) we observed that in 48 hpf embryos npas4a mRNA is expressed exclusively in the brain (Figure 2). We observed particularly strong expression in a discrete cluster of npas4a-expressing cells in the forebrain where they were symmetrically arranged in two parallel rows either side of the midline (Figures 2A–C). No staining was observed in negative control experiments where a sense probe was used (Figures 2D–F).

### Expression of npas4a mRNA is enhanced by neuronal activity

The brain-specific expression pattern of the zebrafish npas4a gene more closely resembled that of the mammalian Npas4 genes than the invertebrate homologs which suggested that the mechanisms underlying its regulation may be similar to those found in mammals. It is known that the rodent Npas4 genes are immediate-early genes (IEGs) that are rapidly up-regulated in response to excitatory synaptic activity (Xiang et al., 2007; Lin et al., 2008; Bepari et al., 2012a,b). We therefore decided to investigate whether the zebrafish npas4a gene is similarly regulated by neuronal activity by treating zebrafish embryos with the γ-aminobutyric acid (GABA) receptor antagonist PTZ (Huang et al., 2001), a seizure-inducing drug which has been shown to activate Npas4 expression in mice (Flood et al., 2004) and which causes up-regulation of activity-dependent IEGs in zebrafish (Baraban et al., 2005; Baxendale et al., 2012).

**Figure 3:**
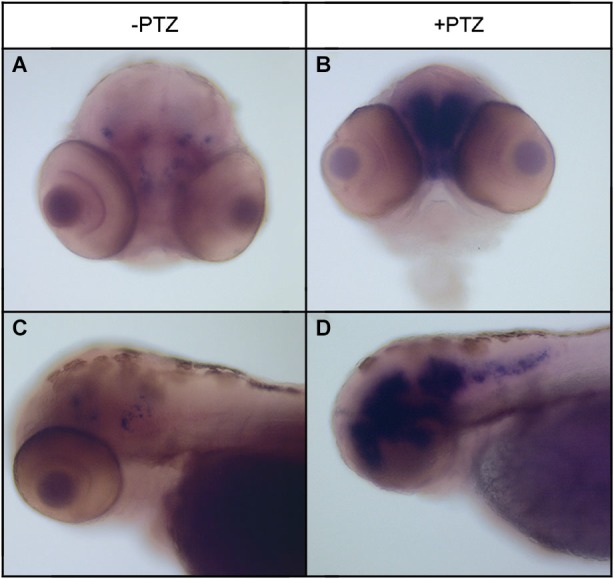
**Expression of npas4a mRNA in 72 hpf zebrafish embryos following PTZ treatment**. Seventy-two hpf embryos were placed in embryo medium containing 3.3 mM PTZ for 20 min after which they were allowed to recover in fresh embryo medium for 30 min before being fixed for whole-mount ISH analysis using an antisense npas4a probe **(B,D)**. Control embryos were treated as above but were placed in embryo medium that did not contain PTZ **(A,C)**. Representative anterior **(A,B)** and lateral views **(B,D)** are shown.

**Figure 4:**
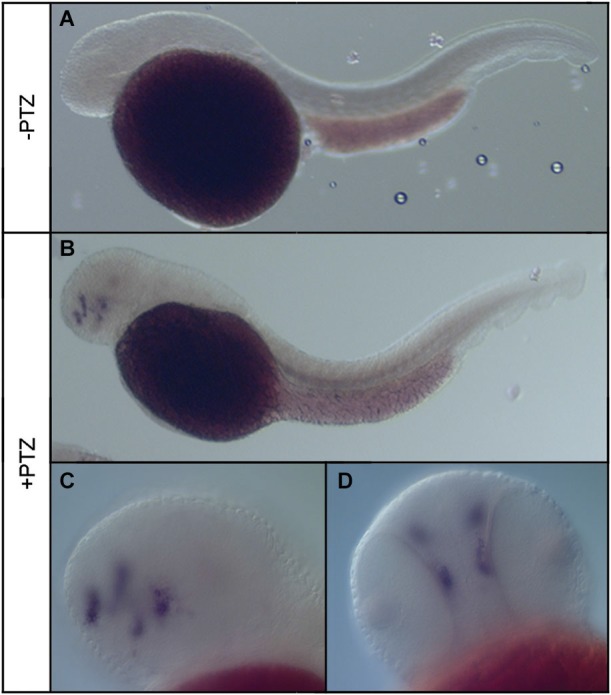
**Expression of npas4a mRNA in 24 hpf zebrafish embryos following PTZ treatment**. Twenty-four hpf embryos were placed in embryo medium **(A)** or embryo medium containing 3.3 mM PTZ **(B–D)** for 20 min after which they were allowed to recover in fresh embryo medium for 30 min before being fixed for whole-mount ISH analysis using an antisense npas4a probe. Representative lateral **(A–C)** and ventral **(D)** views are shown.

To determine the effect of PTZ treatment on npas4a expression, 72 hpf zebrafish embryos were placed in embryo medium containing 3.3 mM PTZ for 20 min and were then allowed to recover in fresh embryo medium for 30 min at which time they were fixed for ISH analysis. Control fish were treated as above but were placed in embryo medium that did not contain PTZ. A marked increase in both the intensity and distribution of npas4a mRNA was observed in PTZ-treated fish compared to untreated controls (Figure 3). At 72 hpf, endogenous npas4a mRNA expression remains confined to the embryonic brain where it is localized in a number of discrete forebrain nuclei (Figures 3A,C). The pattern of PTZ-induced npas4a expression was similar but considerably more intense and also extended further in the posterior direction (Figures 3B,D). Moreover, although endogenous expression of npas4a mRNA was undetectable in 24 hpf embryos under normal conditions (Figure 4A), treatment of embryos with PTZ at this stage induced a strong up-regulation of npas4a expression such that it could be readily detected by ISH in a number of discrete forebrain nuclei (Figures 4B–D).

**Figure 5:**
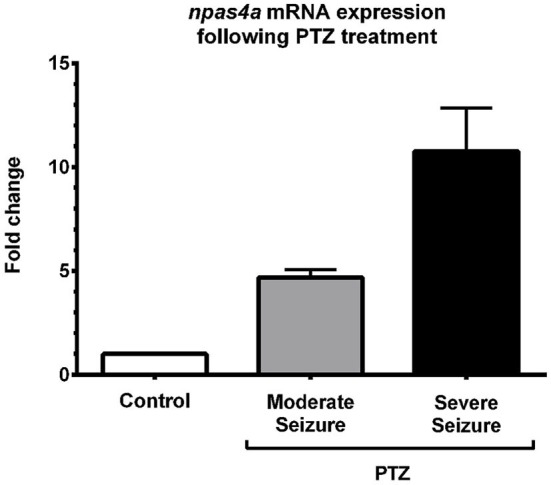
**Quantification of PTZ-induced npas4a expression in the adult zebrafish brain**. One year old zebrafish were placed in embryo medium containing 3.3 mM PTZ for 20 min and were then allowed to recover in fresh embryo medium for 60 min before their brains were removed for RNA extraction. Control fish were treated as above but were placed in embryo medium that did not contain PTZ. Up-regulation of npas4a mRNA expression was quantified using qRT-PCR. For each individual fish, npas4a expression was normalized to actb2 expression. Mean values and standard deviation are displayed (Control, *n* = 8; Moderate seizure, *n* = 3; Severe seizure, *n* = 5). Fold changes are relative to the control group which was given an arbitrary value of 1.

To quantify the increase in npas4a expression following PTZ treatment, we compared npas4a mRNA levels in the brains of control and PTZ-treated adult zebrafish using qRT-PCR. In this series of experiments, 1 year old zebrafish were placed in tank water containing 3.3 mM PTZ for 20 min and were then allowed to recover in fresh tank water for 60 min before their brains were removed for RNA extraction. Control fish were treated as above but were placed in tank water that did not contain PTZ. All of the PTZ-treated zebrafish exhibited convulsive motor activity indicative of generalized seizure (*n* = 8/8). Conversely, no such convulsive activity was observed in untreated animals (*n* = 0/8). Of the PTZ-treated fish that displayed seizure activity, several temporarily lost posture rolling onto their side and became immobile ventral side up for up to 10 s before resuming normal orientation and swimming activity (*n* = 5/8). We interpreted this behavior to be a post-ictal altered conscious state that follows generalized seizure and suggests a more severe level of seizure activity (Cavanna and Monaco, 2009). On account of this we separated PTZ-treated fish into two groups based on whether they experienced moderate or severe seizure activity. Interestingly, we found that PTZ-induced up-regulation of npas4a was commensurate with seizure severity (Figure 5). While npas4a mRNA expression was significantly elevated in the moderate seizure group (4.7-fold increase vs. control group), an even more pronounced increase was seen in the severe group (10.7-fold increase vs. control group). Together these data indicate that, as is the case in mammals, the expression of the zebrafish npas4a gene can be rapidly induced by neuronal activity.

### Knockdown of npas4a during zebrafish development results in increased apoptosis in the brain

**Figure 6:**
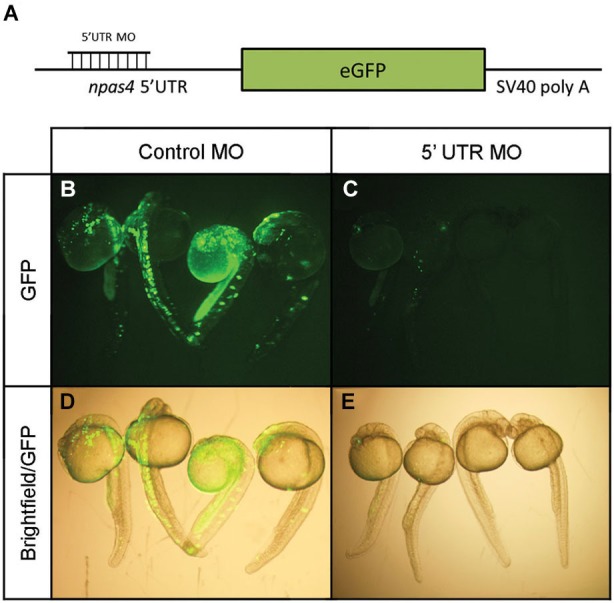
**Verification of npas4a 5′ UTR MO specificity using an eGFP reporter construct. (A)** Schematic representation of the eGFP reporter construct. Single-cell zebrafish embryos were co injected with an eGFP reporter construct which expresses the coding sequence for the eGFP fused to the npas4a 5′UTR and 1.75 pmol of either an antisense npas4a 5′ UTR MO or a mismatched control MO. Expression of eGFP was assessed 24 h after injection in live embryos using fluorescence microscopy. Representative images are shown for control MO **(B)** and 5′ UTR MO **(C)** injected embryos. Fluorescence images are overlaid with corresponding bright-field images to demonstrate areas of eGFP expression **(D–E)**.

In view of the distinctive npas4a expression pattern seen in the developing zebrafish brain, we next investigated the effect of reduced npas4a expression on zebrafish neurodevelopment in a series of loss-of-function experiments. Knockdown of npas4a expression was achieved using injection of antisense MOs targeting the 5′ UTR of the npas4a transcript into the one-cell stage embryo, a technique amenable to this model organism. A mismatch control MO in which five of the nucleotides were randomly substituted was used as a negative control. As no antibody to the zebrafish npas4a protein was available and the antibodies raised to the mammalian Npas4 proteins did not cross-react with the zebrafish homolog (data not shown), an eGFP reporter construct was used to verify the efficacy of the 5′ UTR MO in knocking down transcripts containing an npas4a-specific complementary target sequence (Figure 6A). Co-injection of the 5′ UTR MO with the reporter plasmid, which contains the coding sequence for the eGFP fused downstream of the npas4a 5′UTR, severely diminished the mosaic expression of eGFP that was observed in embryos co-injected with the mismatched control MO and the reporter plasmid (Figures 6B–E).

**Figure 7:**
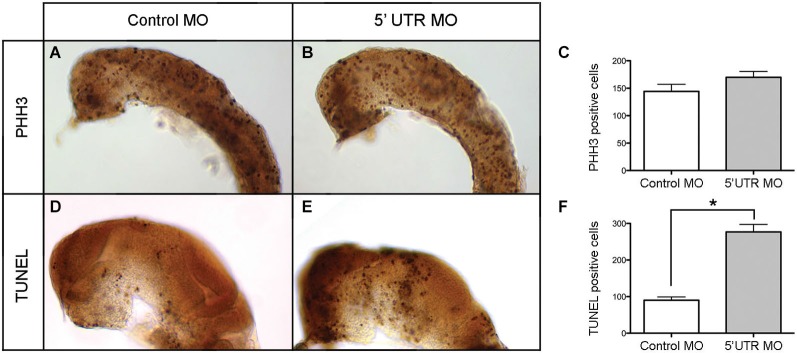
**Quantification of neural proliferation and apoptosis in npas4a morphant and control zebrafish embryos at 24 hpf**. Neural cells undergoing cell division were identified using an antibody to PHH3. Representative images are shown for control MO **(A)** and 5′ UTR MO **(B)** injected embryos. The mean number of mitotic cells **(C)** in the control group was 144 (SEM = 13.1, *n* = 26) while in the 5′ UTR MO group this figure was 170 (SEM = 10.6, *n* = 21). This difference was not statistically significant when analyzed using a 2-tailed *t*-test (*p* = 0.148). The incidence of programed cell death in each group was measured using the TUNEL assay. Representative images are shown for control MO **(D)** and 5′ UTR MO **(E)** injected embryos. The mean number of apoptotic cells **(F)** in the control group was 90 (SEM = 26.4, *n* = 9) whereas in the 5′ UTR MO group this figure was 277 (SEM = 46.2, *n* = 5). This difference was statistically significant when analyzed using a 2-tailed *t*-test (*p = 0.003). Lateral views of a 24 hpf zebrafish are shown with anterior to the left and dorsal up. The skin, eyes and yolk have been removed for greater visibility.

Given the distinct brain-specific expression of npas4a in the zebrafish embryo, we first investigated whether there was any change in the overall cellular composition of the developing brain. To determine whether cellular proliferation in the developing brain is affected by knockdown of npas4a, we assessed the degree of mitotic activity in both control and morphant 24 hpf embryos (Figures 7A,B) using an antibody to phospho-histone H3 (PHH3), a marker of dividing cells (Hans and Dimitrov, 2001). We found that there was no significant difference in the number of PHH3-positive cells between groups (Figure 7C) which implies that cell cycle progression is unaffected by loss of npas4a expression. To address the alternative explanation, we assessed the incidence of programed cell death in each of the groups using the TUNEL assay (Figures 7D,E). We found that there were significantly more apoptotic cells in the brains of embryos injected with the npas4a 5′ UTR MO than the mismatch control MO at 24 hpf (Figure 7F).

### Knockdown of npas4a results in misexpression of forebrain-specific markers

**Figure 8:**
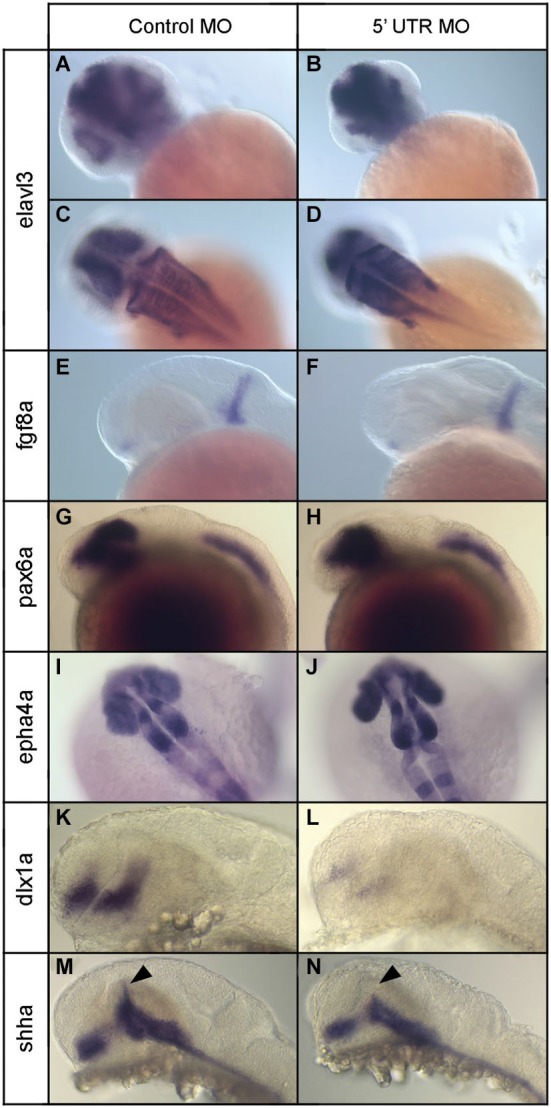
**Expression of various neural marker genes in npas4a morphant and control embryos at 24 hpf**. Embryos were injected with either the control MO **(A,C,E,G,I,K,M)** or the 5′ UTR MO **(B,D,F,H,J,L,N)** and were allowed to develop for 24 hpf prior to whole-mount gene expression analysis. Representative images for a number of neural marker genes are shown; elavl3 **(A–D)**, fgf8a **(E,F)**, pax6a **(G,H)**, epha4a **(I,J)**, dlx1a **(K,L)** and shha **(M,N)**. Note the limited dorsal progression of shha expression in the zona limitans intrathalamica (ZLI) in npas4a morphants (arrowhead).

Next we investigated how the increase in programed cell death seen in npas4a morphants affects neural patterning in the developing zebrafish brain. We first examined the expression of the early neuronal marker elavl3 (Kim et al., 1996; Park et al., 2000) at 24 hpf using immunohistochemistry (IHC) to determine whether reduced npas4a expression has an effect on embryonic neurogenesis. The staining revealed that neurons were still generated in the npas4a morphants in a similar pattern to that seen in control embryos albeit with a noticeable difference in their distribution (Figures 8A–D). The arrangement of neuronal cells was much more condensed in npas4a morphants which is likely due to their decreased head size and thus provides further evidence of gross changes in cytoarchitecture.

To identify the specific brain structures and/or cell populations that are affected, we examined the expression of a range of region-specific neural marker genes at 24 hpf and compared their expression patterns in npas4a morphants and stage-matched control MO-injected embryos. We first analyzed the expression of fgf8a, a gene that encodes a growth factor involved in dorsoventral patterning of the embryo (Figures 8E,F), using ISH. At 24 hpf, expression of fgf8a occurs in two domains; in the anterior neurectodermal ridge and also in a transverse column of expression at the midbrain/hindbrain boundary (MHB; Fürthauer et al., 1997; Reifers et al., 1998). Expression of fgf8a appeared normal in npas4a morphants and the MHB remained intact suggesting expression of npas4a is not involved in patterning this domain.

We then investigated the expression of pax6a, a gene encoding a transcription factor that has regulatory roles in eye development (Nornes et al., 1998) and proliferation of neural progenitor cells (Thummel et al., 2010). At 24 hpf, pax6a is expressed in two domains in the embryo; in the diencephalon, where the posterior edge of pax6 expression demarcates the forebrain/midbrain junction (FMJ), and in a longitudinal posterior domain which begins at the second rhombomere and extends caudally along the neural tube to the tip of the tail (Krauss et al., 1991; Lakowski et al., 2007; Kleinjan et al., 2008). We found no difference in the expression pattern of pax6a between npas4a morphants and control-injected embryos and the positioning of the FMJ appeared normal (Figures 8G,H).

To assess hindbrain development, we used IHC to analyze the expression of epha4a, a segmentation marker that is expressed specifically in odd-numbered rhombomeres (Cooke and Moens, 2002). Hindbrain structure appeared normal and rhombomere boundaries were well defined in npas4a morphants (Figures 8I,J) suggesting that npas4a is not involved in segmentation of the rhombencephalon. We did, however, consistently observe splaying of the retina away from the midline in morphant embryos which is indicative of diencephalon abnormalities.

To further explore the nature of the diencephalic defects present in the npas4a morphant fish, we examined the expression of a several forebrain marker genes that have well-defined expression patterns in the diencephalon. First we investigated the expression of dlx1a which is normally expressed in telencephalon and anterior diencephalon of the forebrain at 24 hpf (Ellies et al., 1997; MacDonald et al., 2010). The dlx1a gene encodes a homeodomain-containing transcription factor that is important for the development of GABAergic interneurons in the zebrafish diencephalon (MacDonald et al., 2013). We found that in npas4a MO-injected embryos, dlx1a expression was markedly reduced in both the telencephalon and diencephalon domains when compared to control MO-injected embryos (Figures 8K,L).

We next examined the expression of the floor/basal plate marker gene sonic hedgehog a (shha). Knockdown of npas4a had a specific effect on the expression of shha. While expression of shha in the floor plate, basal plate and nucleus of the tract of the postoptic commissure (nTPOC) was unaffected, we noted that there was a marked reduction in the dorsal progression of shha expression in the zona limitans intrathalamica (ZLI) at 24 hpf (Figures 8M,N). Together, these results indicate that decreased npas4a expression results in a specific disruption of embryonic forebrain organization and suggest a possible role for npas4a in forebrain patterning.

## Discussion

Here we provide the first detailed characterization of the zebrafish npas4a gene. We show that the zebrafish npas4a protein is evolutionarily more closely related to the mammalian Npas4 homologs than those found in invertebrates, yet it nevertheless forms a distinct branch within the vertebrate lineage which suggests that some divergence or specialization of function may have occurred in fish. Even so, we demonstrate that the regulation and expression of the zebrafish npas4a transcript closely resembles that seen in other vertebrate species. Similarly to the rodent Npas4 genes, the zebrafish npas4a transcript is expressed specifically in the brain, at least until the 72 hpf stage, and is markedly up-regulated in response to excitatory neuronal activity which suggests that there is some conservation of the regulatory mechanisms governing the expression of Npas4 across vertebrate species. Finally, we show that knockdown of npas4a during embryonic development results in forebrain abnormalities and a concomitant reduction in the expression of specific forebrain markers including dlx1a and shha. From this we deduce that zebrafish npas4a homolog is a brain-specific activity-dependent gene that plays a role in forebrain development.

### Expression and regulation of Npas4 is conserved among vertebrate species

Our investigation of npas4a expression in the zebrafish embryo revealed a distinctive, activity-regulated pattern of expression that is most prominent in the ventral forebrain. These observations closely match those reported by others (Baxendale et al., 2012). While no endogenous expression of npas4a was detected at 24 hpf, treatment with the GABA receptor antagonist PTZ at this timepoint induced a robust up-regulation of npas4a mRNA in discrete nuclei of the ventral telencephalon and diencephalon. At 48 hpf, endogenous npas4a expression in these brain regions was evident without the use of convulsive agents. By 72 hpf, the domain of endogenous npas4a expression had expanded to more dorsal parts of the forebrain and midbrain and this expression could be further enhanced by stimulation of neuronal activity using PTZ. Notably, expression of npas4a was restricted to the brain in all of the time-points examined; no expression was observed in other parts of the CNS (i.e., the spinal cord) or in other tissues. Finally, in adult fish, we quantitatively demonstrated that up-regulation of npas4a mRNA in the brain is commensurate with seizure severity.

Two main conclusions can be drawn from our research into the expression of the zebrafish npas4a gene. Firstly, that in the early stages of embryonic development it is expressed exclusively in the brain and, secondly, that it can be rapidly induced by neuronal activity. In this regard, it has more in common with its mammalian homologs, which are activity-dependent IEGs expressed predominantly in the brain (Moser et al., 2004; Lin et al., 2008; Bepari et al., 2012a), than its invertebrate counterparts, which are expressed in tubular structures of the feeding and respiratory systems (Crews, 2003; Jiang and Crews, 2003, 2006). This is in keeping with our phylogenetic analysis of Npas4 proteins and together these findings suggests that, although all of the Npas4 homologs share a common ancestor gene (Jiang and Crews, 2003), there has been some divergence in the regulatory elements controlling Npas4 expression since the branching of vertebrates and invertebrates.

Interestingly, while we were not able to detect endogenous npas4a expression in 24 hpf embryos using ISH, we nevertheless observed specific forebrain phenotypes associated with npas4a knockdown at this time point. A possible explanation for this is that npas4a is expressed at 24 hpf but at levels that are too low to detect using ISH. This is supported by the finding that treatment of embryos with PTZ at 24 hpf enabled npas4a mRNA to be detected, presumably because the low level of basal expression had been up-regulated to the point of detection. If this is the case, then it would seem that even a very low level of npas4a expression is sufficient for normal development. In view of this, it would be of value to explore embryonic forebrain development in a transgenic npas4a null zebrafish strain where there is no endogenous npas4a gene product as this may reveal additional, or more extreme, forebrain phenotypes than those observed in npas4a MO-treated fish.

### The zebrafish npas4a protein plays a role in forebrain development

Our loss-of-function experiments using MOs demonstrated that decreased npas4a expression during zebrafish embryogenesis leads to a specific disruption in the organization of the developing forebrain. While expression of marker genes for the FMJ and the MHB was normal, a number of forebrain abnormalities were observed at 24 hpf including increased apoptosis and altered expression of several forebrain marker genes. We observed reduced expression of dlx1a in both the telencephalon and diencephalon, as well as an impaired ventral-dorsal progression of shha expression in the ZLI of the diencephalon. Interestingly, expression of other markers, such as pax6a and fgf8a, was unaffected, which points to a very specific function for npas4a during forebrain development. Overall, our data show that npas4a is required for proper formation of the forebrain though further investigation is required to clarify its role in CNS development.

### Down-regulation of dlx1a in npas4a morphants

One of the forebrain markers affected by reduced levels of npas4a expression was dlx1a. Knockdown of npas4a resulted in a marked decrease in dlx1a expression in the telencephalon and diencephalon at 24 hpf. This implies that npas4a acts as an upstream regulator of dlx1a expression in the embryonic forebrain though, at present, it is not clear whether this regulation is direct or indirect. Given that dlx1a is an important regulator of GABAergic neuron differentiation in the zebrafish forebrain (MacDonald et al., 2010, 2013), our results suggest that npas4a may also be involved in this process. It should be noted, however, that the existence of paralogous dlx genes with similar functions means that there is often some degree of compensation that occurs when one of the paralogs is lost. Indeed, single dlx gene morphants often display only mild phenotypes on account of this functional redundancy. This is the case for dlx1a and the physically linked paralog dlx2a which share overlapping expression patterns and functions (Ellies et al., 1997; Sperber et al., 2008; MacDonald et al., 2013). Knockdown of either dlx1a or dlx2a alone has no effect on gad1b expression, whereas knockdown of both leads to loss of gad1b expression in the ventral prethalamus and hypothalamus (MacDonald et al., 2013). Since it is not known whether loss of npas4a expression affects only dlx1a or other closely related dlx genes, its impact on GABAergic neurogenesis is difficult to predict. To clarify this, in future studies it will be necessary to investigate the effect of npas4a knockdown on both dlx2a expression and the incidence of GABAergic neurons.

The expression and function of Dlx1 genes is highly conserved among vertebrates and thus our results may also have relevance in other species. As in the zebrafish, the mouse Dlx1 gene is expressed in the developing forebrain (Liu et al., 1997; Eisenstat et al., 1999) where it plays a crucial role in the differentiation and function of GABAergic interneurons (Stühmer et al., 2002a,b; Petryniak et al., 2007). Dlx1−/− mice show an age-dependent loss of specific types of interneurons in the cortex and hippocampus and, as a consequence, they have a reduced level of corticolimbic inhibition which makes them prone to seizure (Cobos et al., 2005). They are also hyperactive and have deficits in fear conditioning (Mao et al., 2009). Interestingly, a number of these phenotypes are also observed in Npas4−/− mice. Specifically, Npas4−/− mice are also hyperactive (Coutellier et al., 2012), prone to seizures (Lin et al., 2008) and also display impaired fear conditioning (Ramamoorthi et al., 2011). Given these similarities, it is tempting to speculate that misregulation of Dlx1 is the common factor that produces similar phenotypes in both mutants though future studies will be needed to address that question. When combined with our results in the zebrafish, these observations support the hypothesis that Npas4 regulates Dlx1 expression and furthermore suggests that this may be a mechanism that is conserved across vertebrates.

Aside from the nervous system, the zebrafish dlx1a gene also has functions in other tissues. From 72 hpf onwards, it is expressed in visceral skeleton and the jaw where is involved in cartilage formation (Verreijdt et al., 2006; Sperber et al., 2008; Talbot et al., 2010). In light of this, it would be of interest to investigate the skeletal structure of npas4a null fish to determine whether they also develop cartilage defects at later stages.

### Misexpression of shha in npas4a morphants

The other forebrain marker gene whose expression pattern was affected by a loss of npas4a expression was shha. The shha gene encodes a diffusible signal molecule that, among other things, is responsible for patterning the thalamic anlange by conferring regional identity to discrete thalamic cell populations. In the zebrafish embryo, expression of shha is first detected at around 60% epiboly and by the onset of somitogenesis it is expressed along the entire rostrocaudal neuraxis. At this stage, expression of shha in the notochord is restricted to the floor plate, while in the brain it is visible as two distinct patches in the diencephalon; anteriorly in the nTPOC and more posteriorly in a region located dorsal to the incipient cephalic flexure (Krauss et al., 1993). As development proceeds, shha expression in these two territories expands, most notably with the formation of the ZLI which begins at around the 22 somite stage (Krauss et al., 1993; Barth and Wilson, 1995; Hauptmann and Gerster, 2000). The ZLI originates from the posterior shha patch via a ventral-to-dorsal progression of shha expression through the alar plate and it becomes fully developed at around 26 hpf when shha expression reaches the alar/roof plate boundary (Scholpp and Lumsden, 2010). The ZLI, also called the mid-diencephalic organizer, is an anatomical structure that separates the prethalamus (anterior) from the thalamus (posterior) but it also functions as a local organizer that directs the development of nearby structures via the secretion of signaling molecules, the principal one being shha (Scholpp et al., 2006).

Unlike dlx1a, where a general decrease in expression was observed throughout the forebrain, knockdown of npas4a resulted in a very distinctive domain-specific reduction in shha expression. Although the ventral domain of shha expression in the floor plate and basal plate was unaffected, we found that loss of npas4a expression specifically prevented the dorsal progression of the shha signal in the ZLI at 24 hpf. It is known that this dorsal diencephalic domain of shha expression in the ZLI is independent of the ventral domain in the basal plate and, furthermore, that it is sufficient for the proper maturation of the diencephalon (Scholpp et al., 2006). This raises the possibility the npas4a specifically regulates the expression of shha in the ZLI and hence that is has a role in patterning the diencephalon. Alternatively, it is possible that reduced npas4a expression leads to an increase in retinoic acid signaling from the epithalamus as it is thought that retinoic acid provides the inhibitory signal that prevents dorsal progression of shha expression (Scholpp and Lumsden, 2010). Interestingly, the failure of npas4a morphants to initiate this dorsal progression of shha expression is reminiscent of the phenotype that is seen when wnt signaling is blocked (Mattes et al., 2012). Moreover, the loss of shha expression in the ZLI of wnt3/wnt3a morphants was associated with increased apoptosis of the organizer cells in this region (Mattes et al., 2012) which is consistent with our observations of increased apoptosis in the forebrain of npas4a morphants. This evidence suggests that there may be an interaction between npas4a and the wnt signaling pathway in the ZLI, or perhaps that npas4a is important for the survival of ZLI organizer cells. These lines of inquiry will need to be investigated further. Nevertheless, our findings suggest a possible role for npas4a in the formation of the ZLI and further support our hypothesis that npas4a plays an important role in forebrain patterning.

Aside from patterning the diencephalon, expression of shha in the ZLI is also important in determining neuronal identity in the thalamus and prethalamus. In particular, the activity of shha as a morphogen influences the glutamatergic/GABAergic fate decision of these neurons and reduced expression of shha can lead to a misspecification of GABAergic neurons (Hagemann and Scholpp, 2012). In light of this, it would be of interest to examine neuronal subtype identity in the diencephalon of npas4 morphants to determine whether the proportion of GABAergic neurons is altered as a result of the irregular shha expression pattern.

### General discussion points

It will be interesting to see whether the findings we present here can be extrapolated to other vertebrate species. To date, almost all of the work relating to the mammalian Npas4 genes has focused on their role in the adult brain and, as a consequence, virtually nothing is known about their expression and function in development. Given the existing similarities between vertebrate Npas4 genes that we have outlined, our results in the zebrafish draw attention to this overlooked area of research by propounding the idea that perhaps the mammalian Npas4 homologs may also have some involvement in CNS development. Interestingly, a recent study demonstrated that the murine Npas4 transcript is also expressed in the developing forebrain (Klaric et al., 2014). While these observations support a possible role for Npas4 in mouse forebrain development, the function of Npas4 in this context remains unknown and is an area that requires further investigation.

Of course, as a result of the whole genome duplication event that occurred in the lineage that gave rise to the teleosts (Taylor et al., 2001; Meyer and Van De Peer, 2005) two npas4 paralogs are present in zebrafish, npas4a and npas4b. Such gene duplication sometimes leads to neofunctionaliztion or subfunctionalization in one or other of the paralogs. It is possible, therefore, that while the regulation and expression patterns are similar between homologs, they may have taken on distinct functions over the course of evolution. Thus, in order to accurately establish the relationship between the zebrafish npas4 genes and their vertebrate homologs, it will be necessary to complete the picture by similarly characterizing the npas4b gene. To date, no data have been published concerning npas4b. Indeed, while it is predicted to encode a protein of 844 amino acids (Bradford et al., 2011), it is not known whether this gene is actively transcribed. Accordingly, characterization of the zebrafish npas4b gene remains a priority for future research.

## Conclusion

In summary, our work shows that the zebrafish npas4a protein is more closely related to other vertebrate Npas4 proteins than invertebrate homologs. We also show that there is considerable similarity in the regulation and expression patterns of the vertebrate Npas4 genes at the transcript level. As in mammals, expression of the zebrafish npas4a gene is activity-dependent and restricted to the brain. Finally, our observation that knockdown of npas4a leads to misexpression of dlx1a and shha highlights a potential role for npas4a in forebrain patterning during CNS development.

The zebrafish, *D. rerio*, is a species with great utility in defining neuro-developmental processes and has many advantages over rodent models due to its rapid external development and transparent embryo. Our work here has shown that research into the regulation and function of the zebrafish npas4a gene is likely to provide valuable insights into the properties of the mammalian Npas4 homologs. We therefore propose that *D. rerio* is a good model organism that can be used to further study the role of this important family of transcription factors in neurodevelopment.

## Conflict of interest statement

The authors declare that the research was conducted in the absence of any commercial or financial relationships that could be construed as a potential conflict of interest.

## Abbreviations

bHLH: basic helix-loop-helix
eGFP: enhanced green fluorescent protein
FMJ: forebrain/midbrain junction
hpf: hours post-fertilization
IEG: immediate early gene
ISH: *in situ* hybridization
MHB: midbrain/hindbrain boundary
MO: morpholino oligonucleotide
npas4a: neuronal PAS domain-containing protein 4
nTPOC: nucleus of the tract of the postoptic commissure
PAS: Per-Arnt-Sim
PTZ: pentylenetetrazol
qRT-PCR: quantitative reverse-transcription PCR
UTR: untranslated region
ZLI: zona limitans intrathalamica.
